# Severe Lactic Acidosis Caused by Thiamine Deficiency in a Child with Relapsing Acute Lymphoblastic Leukemia: A Case Report

**DOI:** 10.3390/children10101602

**Published:** 2023-09-26

**Authors:** Francesco Baldo, Enrico Drago, Daniela Nisticò, Silvia Buratti, Michaela Calvillo, Concetta Micalizzi, Maria Cristina Schiaffino, Mohamad Maghnie

**Affiliations:** 1 Institute for Maternal and Child Health IRCCS Burlo Garofolo, 34137 Trieste, Italy; 2 Department of Neuroscience, Rehabilitation, Ophthalmology, Genetics, Maternal and Child Health (DINOGMI), University of Genoa, 16147 Genoa, Italy; 3 Department of Medicine, Surgery and Health Sciences, University of Trieste, 34127 Trieste, Italy; 4 Neonatal and Pediatric Intensive Care Unit, IRCCS Giannina Gaslini, 16147 Genoa, Italy; 5 Hematology Unit, IRCCS Giannina Gaslini, 16147 Genoa, Italy; michaelacalvillo@gaslini.org (M.C.);; 6 Pediatric Clinic and Endocrinology Unit, IRCCS Giannina Gaslini, 16147 Genoa, Italy

**Keywords:** thiamine deficiency, B1 deficiency, lactic acidosis

## Abstract

Lactic acidosis is characterized by an excessive production of lactic acid or by its impaired clearance. Thiamine deficiency is an uncommon cause of lactic acidosis, especially in countries where malnutrition is rare. We describe the case of a 5-year-old boy who presented with a central nervous system relapse of acute lymphoblastic leukemia. During the chemotherapy regimen, the patient developed drug-induced pancreatitis with paralytic ileus requiring prolonged glucosaline solution infusion. In the following days, severe lactic acidosis (pH 7.0, lactates 253 mg/dL, HCO3- 8 mmol/L) was detected, associated with hypoglycemia (42 mg/dL) and laboratory signs of acute liver injury. Due to the persistent hypoglycemia, the dextrose infusion was gradually increased. Lactates, however, continued to raise, so continuous venovenous hemodiafiltration was started. While lactates initially decreased, 12 h after CVVHDF suspension, they started to raise again. Assuming that it could have been caused by mitochondrial dysfunction due to vitamin deficiency after prolonged fasting and feeding difficulties, parenteral nutrition and thiamine were administered, resulting in a progressive reduction in lactates, with the normalization of pH during the next few hours. In the presence of acute and progressive lactic acidosis in a long-term hospitalized patient, thiamine deficiency should be carefully considered and managed as early as possible.

## 1. Introduction

Lactic acidosis (LA) is a condition characterized by an excessive production of lactic acid or by its impaired clearance; it can be primary or secondary. Primary lactic acidosis is caused by inborn errors of metabolism (IEM), such as disorders of the respiratory chain or of the Krebs cycle, pyruvate dehydrogenase or pyruvate carboxylase deficiency, long-chain fatty acid oxidation disorders, organic acidurias, disorders of biotin metabolism, glycogen storage disease, and gluconeogenesis disorders. Acquired lactic acidosis, on the other hand, may be due to several causes, from the use of a torniquet to obtain a blood sample or a difficult blood draw itself, to severe systemic conditions, like shock, liver or renal failure, sepsis, and intoxications (e.g., ethanol). Thiamine (also called vitamin B1) deficiency is another possible cause of lactic acidosis. Thiamine is a water-soluble vitamin that plays a key role in cellular metabolism, participating in various biochemical pathways. Since it cannot be synthetized endogenously, it must be introduced with foods. Overall, thiamine deficiency (TD) is uncommon in pediatric subjects who live in high-income countries, where malnutrition is rare [1]. In fact, beriberi, as it is also traditionally called, is rather associated with low-income countries, and typically affects infants who are breastfed by thiamine-deficient mothers [2,3]. Malignancies, such as leukemia and neuroblastoma, bone marrow transplant, and total parenteral nutrition or enteral nutrition, singularly or in association, may determine lactic acidosis secondary to TD [4,5,6]. Here, we describe the case of a 5-year-old boy with a relapse of leukemia who developed severe lactic acidosis during chemotherapy complicated by significant toxic pancreatitis.

## 2. Case Presentation

A 5-year-old male patient was referred to the pediatric clinic for right coxalgia and wide-based gait.

His medical history was remarkable for the diagnosis of infant acute lymphoblastic leukemia (ALL) type B at 6 months of life, treated according to the Interfant-99/06 protocol. During maintenance therapy, an isolated central nervous system (CNS) relapse occurred, and the IntReALL high risk protocol was initiated but discontinued early, due to acute toxic leukoencephalopathy. Haploidentical hematopoietic stem cell transplantation (haplo-HSCT) was performed at 2 years of age and was eventually complicated by acute cutaneous and intestinal graft versus host disease (GvHD). The boy was off therapy for approximately 1 year.

At admission, laboratory tests showed an increase in lactate dehydrogenase (LDH) (342 UI/L). A brain MRI showed the diffuse thickening and contrast enhancement of both spinal roots and cranial nerves. The presence of early B-ALL blasts (422 cells, 100% atypical) on cerebral spinal fluid (CSF) associated with negative bone marrow biopsy confirmed the presence of an exclusive CNS relapse. Chemotherapy was started according to the IntReALL SR 2010 protocol, obtaining good clinical, CSF, and radiological responses. However, during the high-risk consolidation HC1 block, the patient developed drug-induced pancreatitis with cholestasis, managed with total enteral nutrition (EN) and octreotide administration. During the following seven days of hospitalization, his clinical condition worsened, and abdominal discomfort appeared due to pancreatitis-related paralytic ileus. Therefore, EN was stopped and replaced with glucosaline solution infusion. At +17 days after the start of the HC1 block, a severe lactic acidosis was detected (pH 7.0, lactates 253 mg/dL, HCO3- 8 mmol/L) associated with ketotic hypoglycemia (42 mg/dL) and laboratory signs of acute liver injury (ammonium 210 umol/L, AST 242 U/L, ALT 357 U/L, bilirubin 7.35 mg/dL, INR 1.8) (Table 1). Plasma amino acid analysis revealed an increase in alanine to 868 umol/L (normal values 185–537), while urinary organic acid analysis showed no abnormalities.

**Figure 1:**
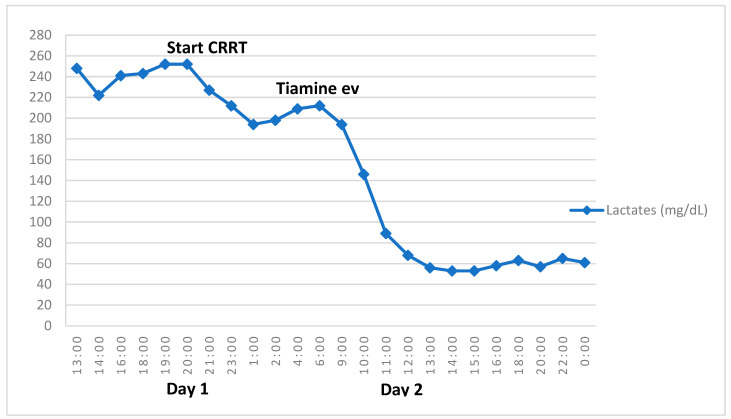
Drop in lactates after thiamine infusion.

Considering the patient’s worsening clinical conditions and comorbidities, he was admitted to the Pediatric Intensive Care Unit (PICU). Due to the appearance of a high respiratory rate without signs of respiratory distress, as well as the persistent lactic acidosis, cardiac failure was suspected. However, his blood pressure and heart rate were normal, as well as his central venous oxygen saturation (80–85%) and diuresis. Low cardiac output syndrome was ruled out after normal echocardiography and chest X-ray. Thus, drug-induced acute liver injury was considered the most probable cause of the patient’s clinical manifestations, and supportive therapy with fresh frozen plasma, packed red blood cells, antithrombin III, vitamin K, and sodium bicarbonate was started. In view of the patient’s comorbidities and blood counts, broad-spectrum antibiotic therapy was started with piperacillin–tazobactam, as well as granulocyte colony-stimulating factor (G-CSF). Considering the patient’s persistent hypoglycemia, the dextrose infusion was gradually increased from a minimum rate of 3.3 mg/kg/min to a maximum of 8.6 mg/kg/min. Lactates, however, continued to raise, so continuous venovenous hemodiafiltration (CVVHDF) was started (Figure 1).

While lactates initially decreased, 12 h after the CVVHDF suspension, they started to increase again. Although thiamine measurement was not available in our laboratory, we assumed that the patient’s biochemical abnormalities could have been caused by mitochondrial dysfunction due to vitamin deficiency after prolonged fasting and feeding difficulties. Therefore, in consideration of the patient’s severe clinical condition, parenteral nutrition and intravenous thiamine (10 mg/kg/day) were promptly administered, resulting in a progressive reduction in lactates with the normalization of pH during the next few hours (Figure 1). Plasma alanine also normalized. The patient discontinued CVVHDF and continued thiamine supplementation for one week, then switched to a standard supplementation of trace elements and vitamins during parenteral nutrition. Following the resumption of oral feeding and a marked improvement in clinical and laboratory conditions (Table 1), the patient was re-admitted to the Onco-Hematology Unit and continued the chemotherapy protocol.

## 3. Discussion

In this report, we describe a case of thiamine deficiency in a child with relapsing acute lymphoblastic leukemia. Severe lactic acidosis led us to the diagnosis of this potentially life-threatening condition, which showed a dramatic response to the intravenous treatment.

Although TD is uncommon in pediatric subjects who live in high-income countries where malnutrition is rare, it should be considered in specific groups of patients, as in our case [1]. Rakotoambinina et al. identified 11 different causes that might be responsible for thiamine deficiency (Table 2) [7].

Didisheim et al. described a male teenager with ALL who developed lactic acidosis and TD deficiency after a parasitic intestinal infection that required prolonged hospitalization and parenteral nutrition, while undergoing chemotherapy [22]. In our opinion, the patient described in our report also falls within this category. Overall, the major role in the development of TD was likely played by the lack of micronutrient intake due to the prolonged use of glucosaline infusion alone. In addition, both the leukemia relapse and the treatment with chemotherapy may have increased the child’s thiamine requirement. In fact, previous studies have shown that cancer cells may develop an alteration in thiamine metabolism. Specifically, hematological malignancies appeared to be particularly prone to developing this metabolic derangement [23]. The exact mechanism, however, is still unknown. It is plausible that, as in breast cancer, abnormal leukocytes may show altered expression of thiamine transporters, increasing intracellular thiamine concentration and enhancing the metabolic pathway connected to it [24].

The complete normalization of both lactate and alanine after thiamine administration is explained by the fact that thiamine acts as a cofactor for pyruvate dehydrogenase. Pyruvate dehydrogenase is a complex of multiple enzymes that converts pyruvate into acetyl-CoA, which is used in the Krebs cycle within the mitochondria to carry out cellular respiration. If pyruvate dehydrogenase does not act properly, as in thiamine deficiency, pyruvate cannot be processed, and it is converted to either lactate or alanine. By administering thiamine, the enzymatic activity was completely restored, hence the incredibly fast response that is reported in Figure 1.

It should be remembered that, apart from thiamine deficiency, there are multiple possible causes of lactic acidosis in patients with malignancies, and they may be seen both in solid tumors and leukemias. The most frequent is the so-called Warburg effect, through which neoplastic cells consume a significant amount of glucose which is later converted into pyruvate and lactate, despite the presence of sufficient oxygen and normal functioning mitochondria (type B lactic acidosis) [25]. The reason why neoplastic cells utilize a less efficient method to produce ATP, instead of consuming glucose through the citric cycle and electron transport as in non-proliferating cells, is only partially understood. However, it is now known that lactic acidosis plays a major role in tumor physiology and aggressiveness, so much so that it may become a valuable target for new antineoplastic treatments [26]. Nonetheless, in patients with leukemias and lymphomas, chronic lactic acidosis unresponsive to the chemotherapy regimen is associated with poor prognosis [27]. Chemotherapy itself may be a cause of lactic acidosis, and few drugs have been associated with this abnormality, especially 5-fluorouracil [28]. Lactic acidosis also has been associated with hepatic and renal metastases in patients with solid tumors [29,30]. However, since most of these reports are outdated, it is unclear whether the lactic acidosis is caused by the metastases per se (impaired lactate clearance) or by the extension and spread of the neoplastic process (increased lactate production, as reported above). Finally, lactic acidosis may also be caused by tissue hypoxia, as in severe infections, whose incidence is higher in immunocompromised subjects, such as those who undergo chemotherapy [31].

From a clinical perspective, it is known that thiamine deficiency can manifest with different patterns in infants, based on their symptoms and the duration of the deficit itself, as reported in Table 3 [1,32].

Remarkably, in this classification, which is a revision of the original one suggested by the World Health Organization in 1999, none of the reported forms recall the pattern developed in our case [33]. For example, cardiac failure, which is commonly associated with lactic acidosis in thiamine deficiency, was absent in our patient. We believe that the onset of severe and progressive lactic acidosis in hospitalized patients, especially if isolated, i.e., in the absence of organ failure and symptoms indicative of other conditions such as sepsis, should suggest the presence of thiamine deficiency. Several reports have been made on this clinical presentation in pediatric subjects in the last two decades, and particularly in those affected by hematological malignancies [5,33,34,35,36]. The common pattern seems to be an overall deterioration of the patient’s general condition (including poor cardiovascular instability, difficulty in extubating, failure to achieve normal laboratory parameters) and a gradual increase in lactate (and thus, of acidosis) despite an increase in the glucose infusion rate. Correctly identifying the clinical and metabolic signs of TD is crucial, especially since thiamine’s assay is relatively complex and may not be feasible in any clinical laboratory, as happened in our case [1].

As for the therapeutic management, the dose and duration of thiamine’s supplementation depends on the severity of the symptoms. In severe cases, the recommended dose is 25–50 mg, given intravenously or intramuscularly for several days, until symptoms disappear. Maintenance is recommended at 2.5–5 mg per day for at least 6 weeks. In mild deficiency, a daily dose of 10 mg of thiamine, followed by 2.5–5 mg a day for 6 weeks, is considered adequate [33].

Retrospectively, a useful information that was underestimated during the diagnostic work-up was the time at which the acidosis appeared. In fact, the half-life of thiamine is usually between 9 and 18 days, which includes the onset of our patient’s symptoms. Another interesting aspect in this case is represented by the partial and brief response to the CVVHDF shown by our patient. The recurrence of lactic acidosis despite hemodiafiltration, as well as the absence of clinical and laboratory signs of sepsis and cardiac failure, should have suggested the presence of an acute biochemical issue at an earlier stage in the patient’s management.

## 4. Conclusions

This case report highlights that, in the presence of acute and progressive lactic acidosis in a long-term hospitalized patient, thiamine deficiency should be carefully considered to manage it as early as possible.

## Figures and Tables

**Table 1:** Patient’s laboratory parameters during his stay in the Pediatric Intensive Care Unit. W5D2, +10 days: week 5, day 1 from the beginning of the IntReALL SR 2010 protocol, +10 days from the beginning of HC1 block. W6D2, +17 days: week 6, day 2 from the beginning of the IntReALL SR 2010 protocol, +17 days from the beginning of HC1 block. W6D5, +20 days: week 6, day 5 from the beginning of the IntReALL SR 2010 protocol, +20 days from the beginning of HC1 block.

Parameter	Unit of Measure	Normal Value	W5D2, +10 Days	W6D2, +17 Days	W6D5, +20 Days
Hb	g/dL	12–14	9.9	10.4	10.0
Ht	%	36–44	28.8	33.6	29.2
MCV	fL	75–95	89.4	99.1	89.7
WBC	×10^3^/uL	3.6–10.7	0.04	0.22	0.82
N	×10^3^/uL	1.79–6.97	0.01	0.03	0.62
L	×10^3^/uL	1.34–5.19	0.03	0.13	0.12
M	×10^3^/uL	0.2–0.76	0.00	0.00	0.06
PLT	×10^3^/uL	150–450	69	20	80
CRP	mg/dL	0–0.46	4.58	1.14	0.74
Glucose	mg/dL	60–100	82	42	112
AST	U/L	0–40	685	242	139
ALT	U/L	0–40	585	357	221
GGT	U/L	11–50		119	283
ALP	UI/L	142–335		81	
Bilirubin					
Total	mg/dL	0–1	5.68	7.35	11.0
Direct	mg/dL	0–0.3	5.05	6.42	9.87
Indirect	mg/dL		0.63	0.93	1.13
INR		0.77–1.23	1	1.8	1.08
Fibrinogen	mg/100 mL	180–350	426	118	236
Ammonium	ug/dL	27–102		210	
LDH	U/L	120–300	666	318	283
Lipase	U/L	13–60	2278	251	505
Amilasi	U/L	0–100	1058	114	129
Albumin	mg/dL	3800–5400	3313	3526	3588
pH		7.31–7.41		7.0	7.39
pCO2	mmHg	41–51		21.4	46.6
Lactate	mg/dL			253	38
HCO3-	mmol/L			8	27.5
BE	mmol/L			−23	6.2

**Table 2:** Causes of thiamine deficiency.

**1**	**Early Infancy Consumption of Thiamine-Free Formula** [8]
**2**	**Inborn errors of thiamine metabolism** Pathogenic variants have been identified in four genes involved in thiamine metabolism. All these inborn errors have specific clinical manifestations: SLC25A2: thiamine responsive megaloblastic anemia (TRMA), which often appears with neonatal diabetic ketoacidosis [9]. SLC25A3: biotin/thiamine-responsive basal ganglia disease, with severe episodes of Leigh-like encephalopathy [9]. SLC25A19: *Amish microcephaly*, which presents with microcephaly, seizures, and episodes of lactic acidosis [9]. TPK deficiency: episodic encephalopathy or early-onset neurodevelopmental delay [9].
**3**	**Diabetic ketoacidosis (DKA)** Urinary thiamine loss is not uncommon in diabetes, and it is believed to worsen the severity of the acidosis [10].
**4**	**Total parenteral nutrition or enteral nutrition** Thiamine deficiency has been reported at various ages, from newborns to adolescents [5,6].
**5**	**Malignancies and bone marrow transplant** [4]
**6**	**Acute critical illnesses** Critically ill patients may suffer from thiamine deficiency due to increased requirement, enhanced loss, or a lack of intake through enteral or parenteral nutrition [11,12,13].
**7**	**Bariatric surgery** [14]
**8**	**Gastrointestinal disorders with impaired thiamine absorption** Short bowel syndrome, inflammatory bowel disease [15,16].
**9**	**Malnutrition in eating disorders** Anorexia and autistic spectrum disorders [17,18,19].
**10**	**High consumption of sugar-sweetened beverages** Most of these cases were reported in Japan [20].
**11**	**Botulism** Secondary to thiaminase producing clostridium botulinum serotype A2 infection [21].

**Table 3:** Different patterns of presentation of pediatric thiamine deficiency [1,32].

Form	Timing	Presentation	
		Early	Late
**Acute cardiologic (Shoshin beriberi)**	1–3 months	Anorexia, restlessness, vomiting	Heart failure signs appear, such as cyanosis and breathlessness
**Aphonic**	4–6 months	Hoarse cry gradually progresses until no sound is produced while the child is crying	Restlessness and edema, followed by breathlessness and death
**Pseudomeningitic**	6–12 months	Nystagmus, bulging fontanelle, convulsions, muscle twitching	Unconsciousness
**Wernicke’s encephalopathy**	Older children/adults	Psychomotor slowing, nystagmus or ophthalmoplegia, ataxia (without is called truncated Wernicke’s encephalopathy), and impaired consciousness.	
**Peripheral neuropathies**	Older children/adults	Pain, tingling, or loss of sensation in hands and feet; loss of deep tendon reflexes; muscle wasting with loss of function or paralysis of the lower extremities; and cranial nerve impairment	

## Data Availability

The authors confirm that the data supporting the findings of this manuscript are available with the paper.

## Abbreviations

LA: lactic acidosis; IEM: inborn errors of metabolism; TD: thiamine deficiency; ALL: acute lymphoblastic leukemia; CNS: central nervous system; HSCT: hematopoietic stem cell transplantation; GvHD: graft versus host disease; LDH: lactate dehydrogenase; MRI: magnetic resonance imaging; CSF: cerebral spinal fluid; EN: enteral nutrition; AST: aspartate aminotransferase; ALT: alanine aminotransferase; INR: international standard ratio; PICU: Pediatric Intensive Care Unit; G-CSF: granulocyte colony-stimulating factor; CVVHDF: continuous venovenous hemodiafiltration.
